# Relation between the Physician and Pharmaceutist

**Published:** 1872-09

**Authors:** 


### EDITORIAL AND MISCELLANEOUS.

---

Relation Between the Physician and Pharmaceutist. As this subject has attracted a good deal of attention of late, we have admitted a discussion of its merits into our columns. In regard to the question raised by Dr. Daniel, of patent medicines, as involving an obstacle in the way of united action between the druggist and physician for an improvement in the laws regulating the sale of drugs, objectionable as the course of the druggists undoubtedly is in this particular, we regret to be compelled to say that this traffic is not only tolerated, but absolutely sanctioned by the Georgia Medical Association, as a number of the members are druggists engaged in the sale of patent medicines, in direct violation of the Code of Ethics of the American Medical Association. We say *sanctioned* because, if we are correctly informed, the question of the exclusion of these individuals was suggested at an annual meeting some years since, and, by either a direct or indirect vote, they were allowed to remain, and are now pursuing this *trade* by the implied authority of the Association. We are satisfied, however, that this action was inadvertently taken, and does not express the real sentiments of the medical profession of the State, and we confidently believe that the Association will remove this discredit to its good name at the next meeting. The Atlanta Academy of Medicine has taken very decided action in regard to this class of medical men.

